# Antimalarial drug prescribing by healthcare workers when malaria testing is negative: a qualitative study in Madagascar

**DOI:** 10.1186/s41182-018-0096-7

**Published:** 2018-04-23

**Authors:** David Harimbola Rakotonandrasana, Takahiro Tsukahara, Noriko Yamamoto-Mitani

**Affiliations:** 1 Centre Hospitalier Universitaire Antananarivo-Hôpital Mère-Enfant Ambohimiandra, 101 Antananarivo, Madagascar; 2 École Doctorale-Nutrition Environnement Santé, Université de Mahajanga, 401 Mahajanga, Madagascar; 3 Department of International Affairs and Tropical Medicine, Tokyo Women’s Medical University, 8-1 Kawada-cho, Shinjuku-ku, Tokyo, 162-8666 Japan; 4 Graduate School of Economics, Hosei University, 2-15-2 Ichigaya Tamachi, Shinjuku-ku, Tokyo, 162-0843 Japan; 5 Department of Gerontological Home-Care and Long-Term Care Nursing, Graduate School of Health Sciences and Nursing, Faculty of Medicine, The University of Tokyo, Hongo 7-3-1, Bukyo-ku, Tokyo, 113-0033 Japan

**Keywords:** Antimalarials, Health personnel, Health policy, Guideline adherence, Drug prescriptions, Qualitative research, Madagascar

## Abstract

**Background:**

Despite the World Health Organization and the National Malaria Program of Madagascar recommending that antimalarial drugs only be prescribed for patients with positive results on malaria rapid diagnostic tests, healthcare workers continue to prescribe these drugs for cases with negative test results. We explored why and how primary healthcare workers in Madagascar continue to prescribe antimalarial drugs despite this guidance.

**Methods:**

We purposively selected 14 medical doctors and 2 nurses from 11 primary health facilities (6 public, 5 private) in Toliara, Madagascar, and interviewed them regarding their antimalarial prescribing behaviors. Semi-structured interviews were conducted, focusing on why and how antimalarials were prescribed for clients with negative rapid diagnostic test results. Interviews were audio-recorded and transcribed verbatim, and the responses were manually coded until consistent themes emerged.

**Results:**

The narrative of healthcare workers regarding their continued prescribing of antimalarials despite negative test results revealed the following: (1) they prescribe antimalarials without positive test results due to their faith to give top priority to clients including the ethical value of beneficence, hope to maintain clinician’s independence, and belief in drug effectiveness; (2) they use antimalarials despite negative test results due to the availability of alternative ways to procure antimalarials; and (3) they carefully select cases to prescribe and determine specific antimalarials despite negative test results by considering the client’s physical condition, preference, and economic status. Our results indicate that healthcare workers prioritized clinician autonomy to give the best care they believed for each client they received, which led to conflict with policy administrators that urged clinicians to follow the national policy and guidelines. Moreover, healthcare workers had access to multiple sources of antimalarial drugs, and there was a lack of consistency in the program provisions that allowed alternative routes for prescribing outside of official policy.

**Conclusions:**

We have shown how a national malarial treatment policy was translated into practice in Madagascar and have highlighted the barriers that may prevent policy success. We must attend to each of these barriers if we are to promote optimal use of antimalarial drugs.

## Background

Parasitological malaria diagnosis, using either a rapid diagnostic test (RDT) or microscopy, has been an essential component of the World Health Organization’s (WHO) guidelines for malaria control [1, 2]. RDTs are preferable in primary healthcare facilities (PHFs) and community settings with limited resources, because unlike microscopy, they are easy-to-use, not time-consuming, and do not require electricity [1]. These advantages mean that RDTs are used in 43 out of 45 African countries in which malaria is endemic [3]. The test-oriented prescribing practice will allow the prevention of not only the wastage of the more efficient but more expensive artemisinin-based combination therapy (ACT) but also of the development of malaria parasite strains, which are resistant to the therapy.

In reality, malaria over-diagnosis and misdiagnosis have continued to be recognized problems in malaria-endemic countries in Africa and Asia [4], and healthcare workers (HWs) at PHFs have continued prescribing antimalarial drugs without positive test results. Several studies have reported that fewer than 50% of HWs adhere to negative RDT results [5–8], although the rate of prescribing antimalarial drug for test-negative cases varies widely (19.0–99.9%) [9]. To benefit from the improved cost-effectiveness of more accurate diagnosis, we need a better understanding of why this over-prescribing behavior occurs.

Unlike in other malaria-endemic countries in Africa, in Madagascar, medical doctors play a principal role in the routine clinical work at PHFs. We previously reported that > 80% of HWs prescribe antimalarial drugs in case of negative test results in Madagascar and that more frequent supervision and experience of frequent RDT positive results in HWs practice have increased their National Malaria Program (NMP) adherence [10]. However, we do not have sufficient knowledge about why and how HWs at PHFs in malaria-endemic regions make treatment decisions for test-negative cases. A qualitative approach can allow us to better understand the perspective of HWs, using which HWs make certain treatment decisions that were not known in previous quantitative studies.

In this qualitative study, we aimed to understand why and how HWs in Madagascar prescribe antimalarial drugs despite negative test results. After gaining more insight into the practice perspectives of these HWs, we then aimed to explore how protocols can be developed for antimalarial drug use and educational programs to promote correct prescribing habits.

## Methods

### Study setting

This study was conducted at regional PHFs in Toliara, the capital of the Atsimo-Andrefana region of Madagascar. This region is characterized as semi-arid with short, occasional, and epidemic-like malaria transmission [11]. The proportion of malaria positive using RDT was 2.4% in this region in 2011 [12].

PHFs are the smallest healthcare system in which qualified HWs (i.e., medical doctors, nurses, or midwives) work in Madagascar. In general, a medical doctor is engaged in routine clinical work as a principal prescriber at PHFs. Health services related to malaria are mainly provided at PHFs by HWs, but they only received outpatients, and patients who need hospitalization are referred to district hospitals.

The District Health Service (DHS) works in collaboration with PHFs to supervise malaria treatment programs and support HWs by giving explanations, praise, or remarks and by updating knowledge. Some HWs at PHFs in the region where this study was conducted received training by the DHS in 2007. Since then, supplementary guidance about managing patients with fevers has occasionally been given to HWs, in written or verbal format, to improve the program. However, there are other programs concerning malaria treatment in Madagascar. In 2006, a separate malaria guideline was launched as a part of the already established Integrated Management of Childhood Illnesses (IMCI) program. These programs not only provide training at medical and nursing schools but also provide continued training to HWs.

### NMP in Madagascar

With some modification for national adaptation, the recommendation of World Health Organization for malaria control [13] led to the development of the new NMP in Madagascar. This program [14], which was launched in 2005, provided all PHFs with multiple strategies for malaria treatment based on the assumption that RDTs are widely available in Madagascar. Specifically, the NMP guidance has recommended that all fever cases (i.e., > 37.5 °C) be tested by RDT or microscopy and that therapy with antimalarials only be prescribed for patients with positive RDT results. In our study setting, parasitological confirmation of malaria was mandatory before prescribing ACT, with the exception that parasite-based diagnosis was not available.

ACT with artemisinin and amodiaquine was the main approach authorized by the government for treating uncomplicated malaria. Artemisinin-lumefantrine was only indicated if this first-line treatment failed. For severe and/or complicated malaria, treatment with quinine injection was recommended. In pregnancy, women who were RDT-positive were prescribed quinine tablets, whereas sulfadoxine-pyrimethamine (SP) was used as intermittent preventive treatment. In the NMP, chloroquine is no longer used for malaria treatment and hence should be withdrawn from PHFs. The NMP has been officially updated in 2007 and 2013 [11, 15]; however, the guideline on antimalarial use has not changed since 2005.

### Availability of antimalarial drugs

In Madagascar, antimalarial drugs are readily available outside governmental healthcare structures, including in private pharmacies and local shops, or from private donations that are beyond governmental control. At the time of the study, several medications were available both in regional services and in private sectors: (1) artemisinin-amodiaquine (government-authorized ACT) was mainly available in PHFs, with limited availability in private facilities; (2) other ACTs (e.g., artesunate-amodiaquine and artemether-lumefantrine) were available at local pharmacies and from community HWs not in the public sector; (3) SP was also available in both public and private health sectors; (4) chloroquine, though officially withdrawn from PHFs, was still available at some private healthcare facilities, local pharmacies, and even local shops; (5) oral quinine tablets were available for pregnant malaria clients in PHFs; and (6) quinine injection was available for severe and complicated malaria in PHFs. Prescribers were free to determine which specific antimalarial drugs to use per case from these options.

The availability of antimalarial drugs has not changed. The latest report of a nationwide study has shown that antimalarials, such as quinine, chloroquine, amodiaquine, and SP, continued to be used, particularly for children aged < 5 years with fever and that the amount of conventional antimalarial use exceeded that of ACTs in 2016 [16]*.*

### Data collection

As we previously reported, 82% (63/77) HWs declared that they prescribed antimalarial drugs in case of RDT negative in the study region [10]. From these 63 HWs, we purposively selected 14 medical doctors and 2 nurses from 11 PHFs. Among the 16 participants, 10 were males. All HWs from private health centers were medical doctors, but from public PHFs, six were medical doctors and two were nurses. Concerning experience, 12 HWs had been qualified for more than 5 years, and only 5 of these had received official training about the NMP (Table 1).

**Table 1:** Characteristics of healthcare workers

Variables	Frequency *N* (%)
Age (completed years)	
31–40	8 (50)
41–50	5 (31.3)
51–60	3 (18.7)
Sex	
Male	10 (62.5)
Female	6 (37.5)
Qualification	
Doctor	14 (87.5)
Nurse	2 (12.5)
Type of workplace	
Public primary health center	8 (50)
Private primary health center	8 (50)
Trained on malaria program/RDT	
Yes	5 (31.3)
No	11 (68.7)
Number of years from graduation	
1–5	4 (25)
6–10	6 (37.5)
11–15	2 (12.5)
> 15	4 (25)
Number of years at the current position	
1–2	5 (31.3)
3–5	8 (50)
> 5	3 (18.7)

*RDT* malaria rapid diagnostic test

The first author (DHR) visited the PHF settings twice, once in December 2008 and once in October 2009, approximately 3–4 years after the NMP had been introduced. DHR is a native speaker of the language used in the interviews. Interviews lasted 20–45 min per participant. The following open-ended questions with follow-up probe questions were used: (1) Why do you prescribe antimalarial for clients without positive RDT results? and (2) How can you prescribe an antimalarial to clients who did not test positive? All interviews were recorded with a digital recorder, transferred to a laptop immediately after the interview, and transcribed within a few days. During the second visit, additional interviews were conducted to gain answers to the questions emerged from the first coding series. Collected data were managed, coded, and categorized in the same way.

### Data analysis

Shortly after the initial visits, the first five interviews were translated into English and coded cyclically by the authors until consistent categories emerged. Those categories were then grouped into themes. When a new theme emerged that was not compatible with existing themes, we created a new one. We continued with the same approach until all data were coded and collated into several themes. We adopted the coding method developed by James Spradley using four analytical procedures: domain analysis, structural analysis, taxonomic analysis, and componential analysis [17]. DHR conducted initial coding, and all authors were involved in the subsequent coding procedures. All codes, categories, and themes were written in English. To ensure confirmability and dependability of our findings [18], all authors regularly met and checked the analyses for consistency and conceptual development as the coding progressed. If there was any disagreement on the analyses, we discussed the matter until we could reach consensus. Furthermore, DHR presented the findings to some interviewees at the second visits to gain credibility. Finally, we tried our best to include the context of NMP practice in Madagascar, which would help the findings to have transferability.

## Results

**Fig. 1:**
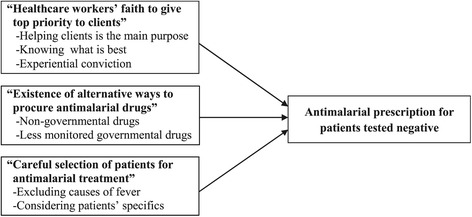
Reasons of antimalarial prescription when malaria testing is negative

Three themes emerged around the issues of HW non-adherence to the NMP guideline on antimalarial drug use: “HW’s faith to give top priority to clients,” “availability of alternative ways to procure antimalarial drugs,” and “careful selection of patients with an indication of antimalarial treatment by HWs.” Each category in the three themes was shown in the Fig. 1.

### HW’s faith to give top priority to clients

There were multiple reasons, depending on the individual context for the prescribers, for why HWs prescribed antimalarial drugs despite the lack of positive RDT results. They firmly believed that they gave the highest priority to clients.

#### Helping clients as the main purpose of practice

HWs considered that their primary duty as a practitioner was to alleviate clients’ suffering and to heal them. They cited that it was their top priority to deliver the best care to their clients in front of them, even if this meant prescribing antimalarial drugs without positive results, because it was the fastest way for clients to recover.

What I’m doing here [for RDT-negative patients], for example a [febrile] child without other obvious cause, I prescribe alternative ACT because I cannot use the government ACT for this case. I tell the mother to come again the next day or something like that, her child is healed, her child is on form, complaints disappeared. What we are searching for is clients’ well-being, isn’t it? (HW 6)

This recognition of duty was considered a principle for all clients, but HWs did comment that private clients or their own relatives received priority and were more frequently treated with antimalarial drugs.

It was the case of my child, my own child, this RDT-negative result happened to him so I did not give antimalarial first but his illness really got worse. I did not support his complaints. Finally, I gave him antimalarials, and he was healed almost right afterward! (HW 5)

For my private clients and even more for my own child, I will not hide from you, I prescribe alternative ACTs even when RDT is negative. (HW 3)

It was also noted that missing cases of malaria may have serious implications for their clients’ health and work. Therefore, prescribing antimalarial drugs based on symptoms was considered the only correct option. If antimalarial drugs were not prescribed, unsatisfied clients may come back many times, complaining about their disease:

I continue to prescribe antimalarial drugs [even with negative RDT]. Even if test result is negative I think it may be malaria and then I will prescribe quinine…You know, I’m worried that if the client is not healed soon he might be laid off by his boss… if he is not healed he should come back again and his boss will say why he should go back many times there? ‘What are you still doing there?’… Do you understand what I mean? (HW 11)

#### We know what is best

HWs said that the final decision to prescribe an antimalarial drug, like any other prescription, rested on them, irrespective of what standardized guidelines or other authorities said. HWs reluctantly and partially followed the guideline that they perceived as a top-down order due to the requests from the Ministry of Health as well as occasional tough enforcement exercised by the regional malaria staff.

That’s clear, it’s just an order … before I denied [the recommendation] because I was not convinced, but a regional malaria staff member came here, … she ‘shouted to me’, that is the right word ‘she shouted to me’...it’s somehow like we do not have right to understand …I asked an explanation and I got this [showing a note from the Ministry of Health]. (HW 9)

It’s curious that it seems that we (doctors) are the worst among educated people, we repeat only what is written in this book [NMP], we are not reasoning at all but following only what is said to do but there are something on which we should use our brain, for this case we should do this, for another case we should do this but not following only what is said inside this book. (HW 5)

Only prescribers had the individualized, detailed information about the lives of each client; put simply, it was claimed that they only knew the client’s perception of the disease and their real need. Indeed, treatment decisions were context-bound, with HWs deciding the treatment appropriateness on a case-by-case basis. HWs perceived that official guidelines failed to allow for this reality of practice:

They [malaria program staff] implement policy, which is their work; we are technicians, we are using techniques [meaning that whatever policy requests, only the prescribers find what is good for clients]. (HW 7)

Clients often presented after self-treatment with an unknown dose of an unknown antimalarial drug for unknown durations, then HWs would question the reliability of RDT-negative results. Moreover, given the limited possibility of optimal follow-up, once the client went home, HWs wondered whether or not treating test-negative clients was truly safe. In such situations where healthcare was not performed in an ideal way, the HWs believed that only they could evaluate the individual needs of each client. The HWs’ concern about missing cases of malaria, which may quickly become complicated, pushed them to favor prescribing antimalarial drugs before the disease “opens out” (HW 12)

#### Experiential conviction

Antimalarial use was also justified based on accumulated, successful experiences in their career. For HWs, prescribing an antimalarial has been the most commonly used and most successful way to manage cases of fever. One HW stated:

Since I started working, presumptive diagnosis alone has been used, and I see that this way to diagnose has been good for clients. (HW 5)

HWs also believed in other advantages of antimalarial drugs, like the anti-inflammatory and antipyretic effects, which they had learned from their own experience or from colleagues. It was reported that a less-than-standard dose of quinine was prescribed for this purpose:

“For this matter, the reason I prescribe antimalarial drugs…when it [RDT] is negative I don’t think any more to malaria but I use it for the anti-inflammatory effect of quinine because I heard that, so the reason I prescribe it [quinine] was to reduce quickly the fever but not for malaria… that is my idea.” (HW 14)

“I want to mention one more thing about the reason of using quinine. We have quinine in stock, and they are there to be used, if we don’t use them, they will just reach their expiration date, so I use them because of their anti-inflammatory effects.” (HW 15)

### Availability of alternative ways to procure antimalarials

No HWs reported the use of government-approved ACT (artemisinin-amodiaquine) without a positive RDT result because medical records of government-approved ACT prescriptions and RDT test results performed at their PHFs were strictly monitored by DHS. However, regardless of whether HWs were working at private or public PHFs, they could prescribe antimalarial drugs both inside and outside their workplace. For example, it is possible to obtain antimalarial drugs outside PHFs, particularly at local pharmacies, where prescribers from PHFs can send their clients to procure alternative antimalarial drugs.

#### Using non-governmental drugs

It is not rare for HWs to prescribe ACTs like artemether-lumefantrine or artesunate-amodiaquine for clients who tested negative. For these ACTs, there is no explicit restriction on their use in the NMP. HWs prescribed them because “Not explicitly forbidden’ means it is allowed to use, doesn’t it?” (HW 12)

#### Using less monitored drugs

Several antimalarial drugs, other than the approved ACT combination, were stored under different levels of strictness but were still available. For example, although both SP and quinine tablets were supplied to PHFs on the basis that they would be used for pregnant women, HWs could order and stock more numbers of these drugs than those expected for practical use because the status of supply and use of these drugs was not strictly monitored. The remaining antimalarial drugs could easily be used in place of ACTs if patients had a negative test result. A HW said “These antimalarial drugs were for what if not clients?” (HW 15) when referring to the large stock available at hand. Some HWs continued to prescribe SP or quinine outside their indications, but others started to partially follow the indications set out by the NMP.

… SP is available but we do not use them. SPs are for pregnant women only. We do not use them for patients. (HW 3)

As for SPs, I do not use them for curative treatment. They are only for pregnant women. I do not use them. (HW 6)

That is the rule already set out; every time RDT is not positive, there should not be any more antimalarial prescription, neither SP nor quinine, that is the rule. For us, SPs are only for women attending antenatal visits, but I prescribe some [for my patients]. (HW 15)

### Careful selection of patients with an indication of antimalarial treatment by HWs

HWs did not prescribe antimalarial drugs to everyone with negative test results. Prescribing antimalarials for RDT-negative patients was not just a way to simplify their job. Rather, there was a specific population for whom they chose to prescribe antimalarials. Each prescriber had accumulated experience on the use of antimalarial drugs and carefully chose what to prescribe according to the client’s need, including consideration of the client’s physical condition, preference, and economic status.

#### Causes of fever excluded

HWs employed standard diagnostic reasoning to decide what antimalarial to prescribe, and how and when a client presents with fever. This included collecting information, considering test results, and deciding whether guideline criteria were met. When only “isolated fever” was found, or when febrile clients had typical signs of malaria (e.g., headache, vomiting, shivering), HWs often prescribed antimalarial drugs. By contrast, most HWs did not prescribe antimalarial drugs when the test results were negative and there were other symptoms like cough, rhinorrhea, or pharyngitis:

My decision [to prescribe antimalarial drugs or not] is based on symptoms. Particularly, when I don’t find any other explanations for fever, I prefer to consider that malaria may be there. For me, the priority is clients’ well-being: Does the child cough? No! Does the child have diarrhea? No! There is no identifiable cause for fever, but RDT is negative… then I may prescribe antimalarials. (HW 13)

Every time I see a case of fever with backache and something like that… I think I go directly to [the diagnosis of] malaria [and prescribe antimalarial drugs]. (HW 11)

The clients’ temperature also affected the choice of antimalarial. Oral medications (e.g., SP tablets, quinine tablets, and chloroquine tablets or syrup) were prescribed when the clinical situation was considered less risky (i.e., lower temperatures). However, when there appeared to be a severe disease, with a temperature around 40 °C, the parenteral route (quinine injection) was preferred. Oral tablets were also converted to parenteral injections if the response to oral therapy was poor. In addition, client preference for injection or oral tablets influenced the HWs choice of route for quinine administration.

#### Looking at specifics

Treatment history influenced antimalarial drug prescribing, with or without antibiotics. HWs stated that they may use antimalarial drugs despite a negative RDT result if a client has persistent fever despite previous treatment with antibiotics. In contrast, patients’ failure to recover with antimalarial alone eventually leads to the addition of antibiotics. They also reported that a recent trip to a high-endemic region would increase the likelihood of prescribing an antimalarial. Many children were also given antimalarial drugs despite negative test results:

We practice according to each case … for children with fever, I prescribe antimalarial drugs … that’s why I said “according to each case” … if clients have signs of sore throat, I don’t prescribe [antimalarial drugs]. (HW 07)

By contrast, where there was a regular and continuous use of insecticide-treated bed nets, this limited antimalarial prescribing. Other test results (e.g., microscopy, Widal test for typhoid fever) were also used to inform antimalarial prescribing practices, and the adverse effects of antimalarial drugs were of serious concern to the HWs. For example, more frequent side effects and intolerance were reported with amodiaquine-based ACTs compared with the other medications. Finally, HWs also considered a client’s economic status, specifically prescribing ACTs sold at very reasonable prices through social marketing if clients had a poor economic status; the more expensive ACTs (e.g., artemisinin-lumefantrine) were only prescribed for those who could afford them. Thus, HWs took multiple client factors into consideration when determining what to prescribe.

## Discussion

In this study, we examined why and how HWs in PHFs did not adhere to the national prescribing guideline set out by the NMP. The reasons for non-compliance with the guideline can be categorized into prioritizing client beneficence, clinician independence for prescriptions, and belief in the effectiveness of antimalarial drugs. In case of negative RDT results, both clinical and non-clinical factors influenced case and antimalarial drug selections. This study highlights issues in translating national policy into individual clinical practice and emphasizes the need to develop a policy that is congruent to the mindsets and working contexts of practitioners for effective policy implementation. This is one of the first attempts to elucidate the experience of prescribers that mostly comprised physicians, unlike some other studies in which non-physician prescribers were interviewed [19, 20].

HWs justified their prescribing behavior by clinician autonomy based on the ethical principle of client beneficence. Antimalarial drugs were used to reinforce their belief that this was appropriate. HWs considered that clinician independence had priority over national policy with respect to client beneficence. A review of clinicians in developed countries indicated that one third considered that guidelines reduced their autonomy and oversimplified medicine [21]. The belief that clinicians should retain independence could be a potential barrier to guideline adherence. It is important for policymakers to consider and emphasize such clinical perspective when defining the efficacy, effectiveness, and efficiency of RDT-based diagnosis and treatment. This relationship between clinician autonomy and over-prescription has rarely been explored in previous literature; in contrast, reports have revealed that clinicians are more passive, being influenced by patient preference, peer pressure, and fear of misdiagnosis [19].

The concept of evidence-based guidelines for clinical practice was originally advocated for informed patient decision-making and rational social judgments, where practical guideline was defined as “systematically developed statements to assist practitioner and patient decisions about appropriate health care for specific clinical circumstances” [22]. Our study revealed that policy administrators urged HWs to adhere the national policy without a detailed explanation of its concept and objective to HWs. Clinicians, policy administrators, and policymakers should have a consensus through dialog that the national policy and guidelines also have every respect for client beneficence. Further, to avoid conflict between clinician independence and guidelines, policymakers should take care to distinguish the scope of a guideline and the range of discretionary decisions available to the clinicians. Past experience of antimalarial use before RDTs has led to strong beliefs in the continued use of the drug among HWs. This is consistent with the results from other studies [23, 24]. Additional beliefs about the drug benefits, including anti-inflammatory and antipyretic effects, justified their continued use. The origin of this belief was unclear, but it seemed to be strengthened by the shared experience.

Although low clinical risk has been demonstrated when not prescribing antimalarial drugs for test-negative cases [25], not offering malaria treatment for false-negative cases might also be inappropriate because the conversion rate from negative to positive RDT can be high [26]. The effectiveness of the RDT-based strategy is sensitive to malaria endemicity [27]*.* Consequently, letting HWs know of the endemicity in the region may be an important strategy. The issue is even more important as malaria incidence is dramatically decreasing during the last 10 years in many parts of the world including Madagascar, and less febrile illnesses are due to malaria [3].

According to the interviewees, there remains a large antimalarial stock in health facilities, and different types of antimalarials, including non-governmental ACTs, are easily available outside health facilities. Addressing the control of antimalarials both inside and outside PHFs should be a priority if the goal of limiting excess antimalarial use is to be realized. Ideally, all medications should be closely monitored at both PHF and pharmacy levels. Accurate information about the numbers of pregnant women expected to attend PHFs should also be monitored to ensure adequate, but not excessive, supplies of SP. Especially, it may be difficult to control antimalarial drugs at private PHFs, and it remains a significant issue.

It may be important that HWs receive further training about the characteristics of non-malarial illnesses, other available tests, correct and updated information on malaria epidemiology, and possible harms of using non-ACTs, in addition to the NMP protocol. Understanding and anticipating the course of non-malarial febrile illnesses could reassure HWs that it is appropriate not to give antimalarials. Additional education on other available tests, such as full blood count, C-reactive protein, urine strip test, and/or Widal reaction, or at least teaching regular diagnostic assessment process, should also be helpful. It should also be educated that the incidence of malaria has been decreasing in Madagascar [16]. Therefore, HWs need to be kept informed so that they can have realistic expectations of the possibility of a febrile patient having malaria.

Lastly, it may also be important that policymakers more clearly understand the clinicians’ perspective, appreciating their willingness to provide best possible care for individual patients that they handle. By mutually understanding each other’s perspective, a good balance can be achieved between individual benefits and those of the society.

We identified the limitations of this study. First, this study has been conducted in a specific setting with one defined malaria strata in Madagascar. Therefore, the generalization of the findings of this study to other malaria-endemic settings is limited. Second, our interpretation was derived from the analysis of translated transcripts, with possible loss of specific cultural meanings and nuances. However, the fact that interviews were conducted, transcribed, and translated by the first author who is a native speaker of the language used during interviews and that regular discussions were conducted between coauthors throughout the study may limit such a loss. Third, our study did not focus on the interaction between clinicians and patients. There may be a tension between HWs and patients which can influence HWs’ prescribing behavior. Further study on the interaction between HWs and patients including interviews from patients is necessary. Fourth, HWs may have changed the prescribing behavior for RDT-negative cases since this study was conducted in 2008–2009. Though the latest formal information on prescribing behavior related to RDT results in the study site was not available, a recent study in Madagascar pointed out HWs’ lack of trust on RDT results and prescribing quinine for RDT-negative patients [28]. To update our knowledge, an evaluation on the current HWs’ adherence to RDT results is needed. Finally, this study did not explore HWs’ behavior when they prescribed concomitantly antimalarial and antibiotic for fear of insufficient treatment. It would be useful to further investigate whether such behavior and perception is the reason for over-prescription.

## Conclusions

Although the guidelines by the WHO and NMP require that only RDT results inform antimalarial prescriptions, complete adherence is difficult to achieve in Madagascar’s primary care context for of a multitude of factors. Notable among these is the basic tension between the HWs autonomy for clinical practice versus the adherence to national policy. However, justification based on client beneficence was compounded by the fact that antimalarial procurement was relatively easy in most situations, indicating that efforts to modify both factors are needed if WHO and NMP guidance is to be realized. Interventions may include giving more information to HWs, educating HWs, limiting the availability of antimalarial drugs, monitoring the prescribing of antimalarials more closely, and ensuring consistency among the different guidelines. Practitioners rightly place the safety of the patient in front of them as their top priority; therefore, guidelines need to be tailored to the contexts in which they will be delivered, taking care to ensure that the concerns of practitioners are addressed.

## Abbreviations

ACT: Artemisinin-based combination therapy
DHS: District health service
HW: Healthcare worker
IMCI: Integrated Management of Childhood Illnesses
NMP: National Malaria Program
PHF: Primary health facility
RDT: Malaria rapid diagnostic test
SP: Sulfadoxine-pyrimethamine
WHO: World Health Organization
